# Association between early bacterial carriage and otitis media in Aboriginal and non-Aboriginal children in a semi-arid area of Western Australia: a cohort study

**DOI:** 10.1186/1471-2334-12-366

**Published:** 2012-12-21

**Authors:** Wenxing Sun, Peter Jacoby, Thomas V Riley, Jacinta Bowman, Amanda Jane Leach, Harvey Coates, Sharon Weeks, Allan Cripps, Deborah Lehmann

**Affiliations:** 1 Division of Population Sciences, Telethon Institute for Child Health Research, Centre for Child Health Research, The University of Western Australia, PO Box 855, West Perth, WA, 6872, Australia; 2 Department of Microbiology & Infectious Diseases, PathWest Laboratory Medicine (WA), Perth, Western Australia, Australia; 3 Microbiology & Immunology, The University of Western Australia, Perth, Western Australia, Australia; 4 Ear Health Research Program, Child Health Division, Menzies School of Health Research, Charles Darwin University, Darwin, Northern Territory, Australia; 5 University Department of Otolaryngology, Head and Neck Surgery, The University of Western Australia, Perth, Western Australia, Australia; 6 Professional Hearing Services, South Perth, Western Australia, Australia; 7 School of Medicine, Griffith University, Gold Coast, Queensland, Australia

**Keywords:** Otitis media, Aboriginal, *Streptococcus pneumoniae*, *Haemophilus influenzae*, *Moraxella catarrhalis*

## Abstract

**Background:**

*Streptococcus pneumoniae* (Pnc), nontypeable *Haemophilus influenzae* (NTHi) and *Moraxella catarrhalis* (Mcat) are the most important bacterial pathogens associated with otitis media (OM). Previous studies have suggested that early upper respiratory tract (URT) bacterial carriage may increase risk of subsequent OM. We investigated associations between early onset of URT bacterial carriage and subsequent diagnosis of OM in Aboriginal and non-Aboriginal children living in the Kalgoorlie-Boulder region located in a semi-arid zone of Western Australia.

**Methods:**

Aboriginal and non-Aboriginal children who had nasopharyngeal aspirates collected at age 1- < 3 months and at least one clinical examination for OM by an ear, nose and throat specialist before age 2 years were included in this analysis. Tympanometry to detect middle ear effusion was also performed at 2- to 6-monthly scheduled field visits from age 3 months. Multivariate regression models were used to investigate the relationship between early carriage and subsequent diagnosis of OM controlling for environmental factors.

**Results:**

Carriage rates of Pnc, NTHi and Mcat at age 1- < 3 months were 45%, 29% and 48%, respectively, in 66 Aboriginal children and 14%, 5% and 18% in 146 non-Aboriginal children. OM was diagnosed at least once in 71% of Aboriginal children and 43% of non-Aboriginal children. After controlling for age, sex, presence of other bacteria and environmental factors, early nasopharyngeal carriage of NTHi increased the risk of subsequent OM (odds ratio = 3.70, 95% CI 1.22-11.23) in Aboriginal children, while Mcat increased the risk of OM in non-Aboriginal children (odds ratio = 2.63, 95% CI 1.32-5.23). Early carriage of Pnc was not associated with increased risk of OM.

**Conclusion:**

Early NTHi carriage in Aboriginal children and Mcat in non-Aboriginal children is associated with increased risk of OM independent of environmental factors. In addition to addressing environmental risk factors for carriage such as overcrowding and exposure to environmental tobacco smoke, early administration of pneumococcal-*Haemophilus influenzae* D protein conjugate vaccine to reduce bacterial carriage in infants, may be beneficial for Aboriginal children; such an approach is currently being evaluated in Australia.

## Background

Otitis media (OM) is one of the most common diseases of childhood [1]. Otitis media with effusion (OME), recurrent acute otitis media (AOM) and chronic suppurative otitis media (CSOM) can lead to impaired hearing which in turn can affect school performance and hence social circumstances in adulthood [2]. An estimated 10-20% of children suffer ≥ 3 attacks of OM in the first year of life in first world settings [3]. Among Aboriginal Australians, OM begins at a younger age and the prevalence of OM and serious complications (in particular CSOM) is higher than in non-Aboriginal children [4,5]. By 14 years of age Aboriginal Australians can expect to have suffered an average of 24 months of OM in contrast to 3 months in non-Aboriginal children [6]. Our own study conducted in the Kalgoorlie-Boulder region of Western Australia (WA) found the peak prevalence of OM was 72% at age 5–9 months in generally asymptomatic Aboriginal children and 40% at age 10–14 months in non-Aboriginal children [5]. Furthermore, on routine examination, 60% of generally asymptomatic Aboriginal children had hearing loss >25 decibels at 6–24 months of age compared with 22% of non-Aboriginal children [5].

*Streptococcus pneumoniae* (Pnc), nontypeable *Haemophilus influenzae* (NTHi), and *Moraxella catarrhalis* (Mcat) are the most important bacterial pathogens associated with OM [4,7-9]. Upper respiratory tract (URT) carriage rates of these pathogens are higher in Aboriginal children than in non-Aboriginal children and onset of bacterial colonization occurs soon after birth in Aboriginal children [4,9,10]. In our Kalgoorlie study, carriage rates of Pnc, NTHi and Mcat at 1–3 weeks of age were 18%, 12% and 17%, respectively, in Aboriginal children and 3%, 2% and 6% in non-Aboriginal children; carriage rates of *Staphylococcus aureus* (Sa) were high in both Aboriginal (55%) and non-Aboriginal (61%) children [9].

Several studies have reported that early onset of URT bacterial carriage increases the risk of subsequent OM. In the USA, children colonized with any of the 3 major respiratory pathogens at age ≤ 3 months were approximately twice as likely to suffer their first episode of OM before the age of 6 months than if colonized later [11]. In a study conducted in a tropical region of the Northern Territory (NT) of Australia involving 2-4-weekly nasopharyngeal carriage and ear assessments in a birth cohort of Aboriginal infants living in a remote area and a birth cohort of non-Aboriginal infants living in an urban area, early dual carriage of Pnc and NTHi was associated with a 33-fold higher risk of OM compared to that in children with early carriage of only one or none of these pathogens [4]. Our Kalgoorlie Otitis Media Research Project (KOMRP) was conducted between 1999 and 2005 to investigate causal pathways to OM [12]. In this study we investigated not only microbiological factors, but also environmental, demographic and socioeconomic factors that might predispose children to OM in order to identify appropriate interventions to reduce the burden of OM [13,14]. Compared with the NT study, we enrolled a larger birth cohort of Aboriginal and non-Aboriginal children, all living within the same broad urban and peri-urban areas in a semi-arid climate and we followed them for 2 years. The availability of information on a broad range of risk factors enabled the investigation of associations between early onset of carriage and subsequent OM over the first two years of life while controlling for potential socioeconomic and environmental confounders. Here we present results on the subset of children participating in the KOMRP who had a nasopharyngeal sample collected during the first 3 months of life and at least one subsequent clinical examination of their ears [12].

## Methods

### Setting and study population

Kalgoorlie-Boulder is a mining town located 600 km east of WA’s capital, Perth. The population is approximately 32,000 with an estimated 8% being of Aboriginal descent [15]. The climate is dry with minimum and maximum average temperatures ranging from 5°C to 18°C and 17°C to 34°C, respectively, and an average annual rainfall of 265 mm [16].

### Study design

A detailed description of methods used in this study has been reported elsewhere [12]. Briefly, 100 Aboriginal and 180 non-Aboriginal children born in Kalgoorlie Regional Hospital between April 1999 and January 2003 and living within an hour’s drive of Kalgoorlie-Boulder were followed from birth to age 2 years. Multiple births, children with severe congenital abnormalities or birth weight < 2000 g and mothers not intending to live in the area for 2 years were excluded.

An initial evaluation was conducted 1–3 weeks postpartum in potential participants’ homes when we sought informed consent and collected a nasopharyngeal aspirate (NPA) to identify bacteria and viruses, demographic data and information on potential risk factors for OM (e.g. smoking in the household, feeding practices, number of people living in the household, number of rooms in the house) using standardized structured interview questionnaires [9,12]. Subsequent follow-ups by research assistants were scheduled at ages 6–8 weeks, and 4, 6, 12, 18 and 24 months and conducted in the project office or in the participants’ homes. During these visits NPAs were collected and we enquired again about time-dependent factors such as feeding practices. Following NPA collection, 1 ml of saline was added to the specimen. A 0.5 ml volume of mucus plug, or, if no visible plug the gently mixed specimen, was pipetted into 1 ml of skim milk-tryptone-glucose-glycerol broth and kept at −20°C for up to 72 hours before transporting to Perth where they were stored at −70°C until processed.

From July 2001 onwards, 7-valent pneumococcal conjugate vaccine (PCV) was offered at ages 2, 4 and 6 months to Aboriginal participants through a national immunization program and we also offered it to our non-Aboriginal study participants.

Details of clinical assessments of children’s ears are described elsewhere [5]. Briefly, an ear, nose and throat (ENT) specialist conducted a clinical examination once before 6 months of age, and again at ages 6–11 and 12–23 months. Diagnosis was established using otoscopy, pneumatic otoscopy and tympanometry, was based on the child’s most severely affected ear, and was classified according to national clinical guidelines at the time [17]. From May 2000 onwards, research assistants also performed tympanometry in children aged 3 months or more at every scheduled routine follow-up visit conducted in the project office or in participants’ homes. All tympanograms were classified by an ENT specialist (HC) or an audiologist (SW) according to standard criteria [2,18]. A type A tympanogram indicates an aerated middle ear, type B the presence of middle ear effusion or a perforation and type C eustachian tube dysfunction. If tympanometry could not be assessed in one ear, then the classification for tympanometry was based on the result for the other ear.

### Laboratory methods

Full details of laboratory methods, including those used to identify bacteria, have been reported previously [9]. Ten microL of each sample was inoculated onto the following media: horse blood agar, chocolate agar containing bacitracin (300 mg/L), vancomycin (5 mg/L) and clindamycin (0.96 mg/L) and colistin nalidixic acid blood agar plates (Oxoid, Australia) [9].

### Data analysis

Data analyses were performed using SPSS (version 15.0 for Windows). Logistic regression models, incorporating generalised estimating equations to account for a loss of independence due to repeated measures on individual children, were used to generate odds ratios to describe the relative likelihood of OM (diagnosed from day of specimen collection onwards) in children who experienced early bacterial carriage compared to children with no early carriage. Limited sample size precluded investigation according to severity of OM. The same analytical methods were used with type B tympanogram as the outcome of interest. All analyses were adjusted for age, age-squared and sex. The age-squared term is included to better capture the observed non-linearity of OM incidence with age in our models. Potential confounders were divided into 2 groups. The first group comprised breast-feeding (exclusive breast-feeding at age 6–8 weeks) and environmental factors i.e. exposure to environmental tobacco smoke (ETS, inside or outside the house), a crowding index (number of people/room) and presence of other children in the house, whilst the second group consisted of simultaneous carriage of other bacteria (Pnc, NTHi, Mcat and Sa as appropriate). Analyses were adjusted for carriage of Sa in view of the high colonization rates with Sa in this population [9] and a possible role of Sa in OM [10,19]. The two groups of confounders were first entered separately into the regression models followed by a final model containing all variables. The crowding index was divided into quintiles for Aboriginal and non-Aboriginal children separately. PCV vaccination was not included as a potential confounder because the first dose of three in the PCV schedule is given at 2 months of age which is too early to have any influence on pneumococcal carriage under 3 months of age. Separate analyses were conducted for Aboriginal and non-Aboriginal children.

The number of otopathogens (Pnc, Mcat, NTHi) identified in children aged <1 month was too sparse to investigate risk of OM associated with carriage in the first month of life. We therefore conducted analyses based on identification of pathogens (a) in one or other or both of the two NPAs collected before the age of 3 months and (b) only in NPAs collected at age 1- < 3 months. As the findings were broadly similar, we present results using bacteriology data obtained at 1- < 3 months.

### Ethical approval

Ethical approval to conduct this study was given by the WA Aboriginal Health Information and Ethics Committee, the Northern Goldfields Health Service and Nursing Education Ethics Committee in Kalgoorlie, Princess Margaret Hospital for Children Ethics Committee and the Confidentiality of Health Information Committee of the Health Department of WA.

## Results

### Study participants

Eighty Aboriginal children had NPAs collected at age 1- < 3 months of whom 66 (83%) had at least one clinical examination by an ENT specialist between the time of NPA collection and age 2 years; 171 non-Aboriginal children had NPAs collected of whom 146 (85%) had at least one clinical examination. A total of 133 clinical examinations were performed between the NPA collection at 1- < 3 months and age 2 years in Aboriginal children (37% before age 6 months, 27% at 6–11 months and 35% at age 12–24 months) and 310 in non-Aboriginal children (32% <6 months, 27% at 6–11 months and 41% at 12–24 months), with a median of two clinical examinations in both Aboriginal and non-Aboriginal children.

### Upper respiratory tract carriage rates

Among members of the KOMRP study population who had NPAs collected at 1- < 3 months and at least one subsequent clinical examination, 60% of Aboriginal children and 14% of non-Aboriginal children carried at least one of the otopathogens (Pnc, NTHi or Mcat) at age 1- < 3 months. The carriage rates of individual pathogens were similar to those reported previously [9]: in Aboriginal children carriage rates of Pnc, NTHi and Mcat were 45%, 29% and 48%, respectively, and in non-Aboriginal children 14%, 5% and 18%, respectively. 17% of Aboriginal children carried both Pnc and NTHi, 30% both Pnc and Mcat, and 18% NTHi and Mcat; 12% carried all 3 pathogens; corresponding figures in non-Aboriginal children were 2%, 6%, 2% and 1% (Table 1). At age 1- < 3 months, Sa was isolated from 39% of NPAs collected from Aboriginal children and 47% of NPAs from non-Aboriginal children.

**Table 1:** **Carriage status at age 1**- < **3 months of *Streptococcus pneumoniae***, **nontypeable *Haemophilus influenzae *and**/**or *Moraxella catarrhalis *and presence or absence of otitis media** (**OM**) **in Aboriginal and non**-**Aboriginal children who had at least one clinical ear examination**

**Micro-organism**			**OM diagnosis**^**a**^			
			**Aboriginal n** **=** **66**		**Non**-**Aboriginal n** **=** **146**	
**Pnc**^**b**^	**NTHi**^**c**^	**Mcat**^**d**^	**Yes (%)**	**No (%)**	**Yes (%)**	**No (%)**
+	+	+	7 (87)	1 (13)	2 (100)	0 (0)
+	+	-	3 (100)	0 (0)	1 (100)	0 (0)
+	-	+	9 (75)	3 (25)	4 (57)	3 (43)
-	+	+	4 (100)	0 (0)	1 (100)	0 (0)
+	-	-	4 (57)	3 (43)	2 (20)	8 (80)
-	+	-	4 (80)	1 (20)	0 (0)	4 (100)
-	-	+	7 (87)	1 (13)	11 (65)	6 (35)
-	-	-	9 (47)	10 (53)	42 (40)	62 (60)

^a^ Otitis media (OM) diagnosed once or more from time of specimen collection at age 1- < 3 months until last clinical examination before 2 years of age.

^b^*Streptococcus pneumoniae*.

^c^*Haemophilus influenzae*.

^d^*Moraxella catarrhalis*.

+ pathogen present.

- pathogen absent.

### Diagnosis of otitis media

OM was diagnosed at least once in 71% (47/66) of the Aboriginal children and 43% (63/146) of the non-Aboriginal children who had an NPA collected and at least one clinical examination (Table 2). Of the 133 clinical examinations recorded in Aboriginal children 40 (30%) were normal, 15 (11%) had a diagnosis of eustachian tube dysfunction (ETD), 63 (47%) had OME, 9 (7%) AOM with or without perforation and 6 (5%) CSOM. The corresponding figures among 310 events documented in non-Aboriginal children were 188 (61%), 31 (10%), 80 (25%), 11 (4%) and 0 (0%). As reported previously for all the KOMRP study participants [5,14], compared with non-Aboriginal children, more Aboriginal children were exposed to ETS, fewer were breast-fed at 6–8 weeks postpartum and they lived in more crowded households (Table 2).

**Table 2:** **Demographic and environmental characteristics**, **and proportion of children with otitis media diagnosed at least once in Aboriginal and non**-**Aboriginal children who had NPA collected at 1**- < **3 months and at least one subsequent clinical examination for otitis media before age 2 years**

**Predictor variable**	**Aboriginal**	**non**-**Aboriginal**	p value^*^
	**n** **=** **66**	**n** **=** **146**	
Male	39 (59.1%)	75 (51.4%)	0.30
OM diagnosed at least once	47 (71.2%)	63 (43.2%)	0.0002
Exposure to environmental tobacco smoke	38 (59.4%)	58 (39.7%)	0.009
Exclusive breastfeeding 6–8 weeks postpartum	31 (47.0%)	94 (64.8%)	0.13
Crowding index (>1 person per room)	46 (69.7%)	19 (13.0%)	<0.0001
Other children living in the house	49 (74.2%)	72 (49.3%)	0.0007

Discrepancies between total numbers of children for different variables are the result of missing data.

^*^ P values are for comparisons between Aboriginal and non-Aboriginal children.

### Bacterial colonization and risk of OM

Table 1 shows the carriage of Pnc, NTHi and/or Mcat individually or concurrently at 1- < 3 months of age in children with and without a diagnosis of OM during the study period. Simultaneous carriage of two or more of these pathogens was very common in Aboriginal children, such that only 23% (n = 7) of all Pnc, 25% (n = 8) of Mcat and 25% NTHi (n = 5) were isolated in the absence of the other otopathogens, in contrast to 50% (n = 10) of all Pnc, 63% of Mcat (n = 17) and 50% (n = 4) of NTHi in non-Aboriginal children. In those who carried Pnc, NTHi or Mcat at age 1- < 3 months, OM was diagnosed respectively at least once in 77%, 90% and 84% of Aboriginal children and 45%, 50% and 67% of non-Aboriginal children. Nine of the 10 children carrying all three otopathogens simultaneously at age 1- < 3 months developed OM, all nine children who simultaneously carried either Pnc and NTHi or NTHi and Mcat developed OM, while 13 (68%) of the 19 children who simultaneously carried Pnc and Mcat developed OM. In contrast, if none of the three otopathogens was isolated at 1- < 3 months of age, 47% of Aboriginal and 40% of non-Aboriginal children were diagnosed with OM on one or more occasions. None of the four non-Aboriginal children who carried NTHi alone developed OM.

Table 3 shows the associations between early carriage of Pnc, NTHi, Mcat or Sa and subsequent risk of OM, first adjusted only for age and sex, then for the presence of the other bacterial pathogens of interest, then for environmental factors, and finally for all potential confounders. When comparing children with early onset of carriage to those without early carriage, the early carriage of any of the otopathogens (Pnc, NTHi and Mcat), either alone or in conjunction with one or both of the others, was associated with an increased risk of subsequent OM in Aboriginal children after adjusting for age, sex and the presence of the other bacterial pathogens. This increased risk declined after adjustment for environmental factors (odds ratio (OR) from 3.68 to 2.44). In non-Aboriginal children, there was a small positive association between early carriage of any otopathogen and subsequent risk of OM, with no difference in odds ratios between the different models; the fully adjusted OR for early carriage and risk of OM was 1.42 (95% confidence interval (CI) 0.78-2.56).

**Table 3:** **Association between carriage of *Streptococcus pneumoniae***, **nontypeable *Haemophilus influenzae***, ***Moraxella catarrhalis *and *Staphylococcus aureus *individually or any of the otopathogens at age 1**- < **3 months and subsequent diagnosis of otitis media adjusting for age and sex**, **presence of other bacteria**, **and environmental factors**

**Adjusted factors**	**Aboriginality**	**Any otopathogen**^**a**^	***S.pneumoniae***	***M.catarrhalis***	***H.influenzae***	***S.aureus***
		**OR (95%CI)**^**b**^	**OR (95%CI)**	**OR (95%CI)**	**OR (95%CI)**	**OR (95%CI)**
**Sex and Age only**	Aboriginal	**3**.**86** (**1**.**61**-**9**.**24**)	1.39 (0.64-3.01)	1.97 (0.89-4.39)	**5**.**32** (**2**.**12**-**13**.**35**)	0.64 (0.26-1.56)
	Non-Aboriginal	1.47 (0.80-2.71)	1.12 (0.50-2.47)	**2**.**57** (**1**.**35**-**4**.**91**)	1.1 (0.36-3.42)	1.02 (0.58-1.77)
**Presence of other bacteria**	Aboriginal	**3**.**68** (**1**.**51**-**8**.**96**)	0.86 (0.39-1.93)	1.72 (0.74-3.96)	**5**.**32** (**1**.**95**-**14**.**49**)	0.6 (0.25-1.47)
	Non-Aboriginal	1.48 (0.80-2.72)	0.77 (0.32-1.88)	**2**.**81** (**1**.**40**-**5**.**61**)	0.96 (0.33-2.83 )	1.06 (0.61-1.86)
**Environmental factors**^**c**^	Aboriginal	2.54 (0.93-6.93)	1.14 (0.49-2.65)	0.81 (0.29-2.27)	**3**.**66** (**1**.**31**-**10**.**25**)	0.54 (0.22-1.33)
	Non-Aboriginal	1.41 (0.78-2.55)	1.07 (0.51-2.28)	**2**.**42** (**1**.**31**-**4**.**48**)	0.87 (0.32-2.39)	1.06 (0.58-1.93)
**All confounders**	Aboriginal	2.44 (0.88-6.78)	0.84 (0.32-2.17)	1.02 (0.34-3.10)	**3**.**71** (**1**.**22**-**11**.**23**)	0.52 (0.20-1.33)
	Non-Aboriginal	1.42 (0.78-2.56)	0.81 (0.33-2.00)	**2**.**63** (**1**.**32**-**5**.**23**)	0.80 (0.30-2.09)	1.05 (0.58-1.91)

All associations adjusted for sex, age and age^2^.

^a^ Any otopathogen includes one or more of *S*. *pneumoniae*, nontypeable *Haemophilus influenzae* and *Moraxella catarrhalis*.

^b^ Odds ratio and 95% confidence interval comparing the odds of diagnosis of OM between those who carried the respective organisms at age 1- < 3 months and those who did not.

^**c**^ Environmental factors are: passive smoking, crowding, other children living in the house and also exclusive breast-feeding at 6–8 weeks.

We then considered individual bacterial pathogens. There was no association between early carriage of Pnc and OM in either Aboriginal or non-Aboriginal children. In non-Aboriginal children, isolation of Mcat at age 1- < 3 months increased the risk of developing OM and there was little change in odds ratios after adjusting for confounders (OR =2.63, 95% CI 1.32-5.23). We found no association between early Mcat carriage and risk of OM in Aboriginal children. In contrast, early NTHi carriage increased the risk of developing OM in Aboriginal children (OR = 3.71, 95%CI 1.22-11.23 after adjustment for all potential confounders) but not in non-Aboriginal children. The OR for increased risk of OM associated with NTHi carriage in Aboriginal children fell from 5.32 (when adjusted for age, sex and presence of the other three pathogens) to 3.71 after adjusting further for environmental factors. Finally, there was a suggestion of a protective effect of Sa carriage in Aboriginal children (OR = 0.52, 95%CI 0.20-1.33 after adjusting for all confounders).

### Tympanometry

Seventy-one Aboriginal children and 160 non-Aboriginal children had had NPAs collected at age 1- < 3 months and tympanometry assessments from age 3 months onwards; 583 assessments (131 in Aboriginal and 452 in non-Aboriginal children) were performed by research assistants at routine field follow-up visits and 342 assessments (99 in Aboriginal children and 243 in non-Aboriginal children) were performed by audiologists at routine ENT clinics. In Aboriginal children 73 (32%), 125 (54%) and 32 (14%) were types A, B and C, respectively; corresponding figures in non-Aboriginal children were 418 (60%), 158 (23%) and 119 (17%). Of the Aboriginal children, 58 (82%) had a type B tympanogram on at least one occasion while 88 (55%) non-Aboriginal children had a type B tympanogram at least once. The median number of tympanometry assessments was three for Aboriginal children and four for non-Aboriginal children.

The same analyses as for a clinical endpoint of OM (using generalised estimating equation regression models) were performed using type B tympanogram as the endpoint, except that we also controlled for the location where tympanometry was done (at field follow-up visit or at the ENT specialist clinic). ORs assessing early carriage and subsequent risk of a type B tympanogram were broadly similar to those found for a clinical outcome of OM, although generally lower (Table 4). In Aboriginal children nasopharyngeal colonization with NTHi at age 1- < 3 months was associated with subsequent type B tympanogram (OR = 5.35, 95% CI = 1.97-14.87) after adjusting for all confounders. In non-Aboriginal children, in contrast to our findings with clinical OM as the endpoint, there was no association between early colonization with Mcat and subsequent type B tympanogram (OR = 1.09, 95% CI = 0.60-2.00).

**Table 4:** **Association between carriage of *Streptococcus pneumoniae***, **nontypeable *Haemophilus influenzae***, ***Moraxella catarrhalis *and *Staphylococcus aureus *individually or any of the otopathogens at age 1**- < **3 months and subsequent type B tympanogram adjusting for age and sex**, **presence of other bacteria**, **and environmental factors**

**Adjusted factors**	**Aboriginality**	**Any otopathogen**^**a**^	***S.pneumoniae***	***M.catarrhalis***	***H.influenzae***	***S.aureus***
		**OR (95%CI)**^**b**^	**OR (95%CI)**	**OR (95%CI)**	**OR (95%CI)**	**OR (95%CI)**
**Sex and Age only**	Aboriginal	2.21 (0.91-5.42)	1.16 (0.55-2.17)	1.60 (0.74-3.48)	**4**.**58** (**1**.**98**–**10**.**59**)	0.61 (0.27-1.39)
	Non-Aboriginal	1.36 (0.84-2.22)	1.59 (0.87-2.91)	1.42 (0.86-2.34)	0.97 (0.32–2.97)	0.92 (0.57-1.48)
**Presence of other bacteria**	Aboriginal	2.07 (0.82-5.22)	0.67 (0.33-1.38)	1.63 (0.79-3.36)	**5**.**51** (**2**.**41**-**12**.**58**)	0.51 (0.24-1.09)
	Non-Aboriginal	1.36 (0.83-2.21)	0.86 (0.29-2.58)	1.28 (0.73-2.26)	1.48 (0.75-2.93)	0.97 (0.60-1.55)
**Environmental factors**^**c**^	Aboriginal	1.56 (0.51-4.74)	0.99 (0.43-2.27)	0.68 (0.23-1.98)	**4**.**31** (**1**.**64**-**11**.**32**)	0.61 (0.25-1.54)
	Non-Aboriginal	1.12 (0.66-1.88)	1.33 (0.75-2.37)	1.16 (0.66-2.05)	0.90 (0.27-3.02)	0.90 (0.52-1.55)
**All confounders**	Aboriginal	1.56 (0.52-4.68)	0.66 (0.32-1.36)	0.99 (0.33-2.93)	**5**.**35** (**1**.**97**-**14**.**87**)	0.45 (0.18-1.13)
	Non-Aboriginal	1.11 (0.60-1.87)	1.31 (0.70-2.43)	1.09 (0.60-2.00)	0.83 (0.24-2.90)	0.92 (0.54-1.58)

All associations adjusted for sex, age, age^2 ^and where tympanometry was conducted (field or clinic).

^a^ Any otopathogen includes one or more of *S*. *pneumoniae*, nontypeable *Haemophilus influenzae* and *Moraxella catarrhalis*.

^**b**^ Odds ratio and 95% confidence interval comparing the odds of having a type B tympanograms between those who carried the respective organisms at age 1- < 3 months and those who did not.

^**c**^ Environmental factors are: passive smoking, crowding, other children living in the house and also exclusive breast-feeding at 6–8 weeks.

## Discussion

To our knowledge, this is the first investigation of early bacterial carriage conducted simultaneously in young Aboriginal and non-Aboriginal children living in the same setting, in this case in an urban and peri-urban rural area of Western Australia. It is one of very few studies investigating associations between early carriage and subsequent OM, despite evidence to suggest that early URT carriage may affect development of long-term humoral and cellular immunity, which in turn can increase susceptibility to disease [20,21].

We have reaffirmed the high rates of single as well as multiple carriage of Pnc, Mcat and NTHi from a young age in Aboriginal children and high rates of OM in both Aboriginal and non-Aboriginal children. Early nasopharyngeal carriage of one or more of the 3 otopathogens, and specifically NTHi, increased the risk of OM in Aboriginal children, while in non-Aboriginal children Mcat was associated with an increased risk of OM as determined by full clinical examination but not by tympanometry alone. The important contribution of environmental factors to the high rates of OM in Aboriginal children [13,14] is emphasized by the observation of reductions in ORs for early carriage (NTHi or any otopathogen) and subsequent risk of OM when controlling for environmental factors (Table 3).

We found no association between early carriage of either Pnc or Mcat and risk of OM in Aboriginal children. This may be because it is only their carriage during the first month (rather than the first 3 months of life) that increases risk of OM, but our data were too sparse to restrict analysis to carriage during the first month of life. Pnc and Mcat carriage begins at a younger age than NTHi: by age 2 months, ~40% of Aboriginal children had carried Pnc and/or Mcat at least once compared with 27% for NTHi [9].

There were similarities and differences between the current study and the only other comparable study which was conducted among Aboriginal children in the NT [4]. Both studies identified the importance of early onset of URT carriage and risk of OM. However, in the NT study Aboriginal children were seen 2-4-weekly from birth and both carriage and ear health status were assessed at each visit. The primary endpoint in the NT study was time to first documented episode of OM (which occurred in all Aboriginal children in the NT before the age of 3 months), while our primary endpoint was any diagnosis of OM up to age 2 years. Carriage rates of single and multiple pathogens were even higher in the NT study than in ours; hence, in the NT study it was more difficult to investigate the contribution of individual otopathogens to subsequent disease than in our present study. Our lower carriage rates are unlikely to be due to differences in laboratory methods as our laboratory scientist (JB) was trained by colleagues in the NT and used the same methods throughout. The most likely explanation for differences in carriage rates between the two study populations is the somewhat better socioeconomic status in our urban/peri-urban WA community than that on a remote island in the NT, also reflected in the higher prevalence of OM and CSOM in the NT community than in our study population [4,5].

The positive association between early carriage of Mcat, but not Pnc or NTHi, and OM in the non-Aboriginal children in our study is consistent with findings in non-indigenous North American children [11]. However, we did not find an association between early Mcat carriage and presence of middle ear effusion as determined by type B tympanogram, possibly because we were identifying milder forms of OM using tympanometry at routine follow-up in the field than in the clinic setting. Of the three otopathogens, Faden *et al*. found that early carriage (defined as <3 months) of Mcat was the most strongly associated with early onset of OM, though the authors also reported that early carriage of any pathogen resulted in earlier onset of OM [11]. In our study, there was a small positive association between early carriage of any pathogen and risk of clinical OM in non-Aboriginal children. The lack of association in non-Aboriginal children between early NTHi carriage and subsequent OM is most likely due to the small number of NTHi isolated before age 3 months (total of 8 of which only 4 were isolated in absence of other otopathogens). Similarly, the lack of association between early Mcat carriage and subsequent OM in Aboriginal children in our study is likely to be the small number (8) of Mcat isolated alone in this population (37% of all Mcat were isolated together with Pncs, 13% with NTHi and 25% with both Pnc and NTHi). This is in contrast to non-Aboriginal children in whom 63% of all Mcat (17/27) were the only otopathogen isolated from an NPA sample. To our knowledge there are no data available to explain in biological terms why one pathogen is more likely to predispose to OM than another.

Our findings suggest that carriage of more than one bacterial pathogen is more likely to result in OM than carriage of a single pathogen (Table 1). However, in view of the small number of isolates detected at an early age and the limited number of subsequent clinical examinations, we were unable to conduct formal statistical analyses to investigate this further. Nevertheless, a strength of our study was that we were able to use type B tympanogram to confirm our analyses using clinical diagnosis of OM as an endpoint.

In addition to the limited sample size, there are several other limitations or potential biases in this study. Firstly, we were not able to conduct a clinical examination every time an NPA was collected due to lack of available specialist personnel, though we were able to conduct tympanometry at the same time as NPA collection from age 3 months onwards. Secondly, in view of the long period of follow-up required, it is possible that we did not enroll children at greatest risk of OM, since we did not enroll children whose families were not intending to stay in the area for 2 years. We previously looked at how representative our KOMRP study population (of which our present data form a subset) was of the general population in the area: there were more teenage Aboriginal mothers, non-Aboriginal parents were older and non-Aboriginal mothers were less likely to smoke in pregnancy than the general population [12].Thirdly, while all children were asked to attend a clinic 3 times over 2 years for routine examination, it is possible that those who attended were sicker children seeking medical attention. Conducting tympanometry during routine field follow-up visits helped address this potential bias. Finally, we cannot exclude the possibility of residual confounding.

The results reported here were part of a broader study investigating causal pathways to OM, allowing us to control for important environmental factors. It would now be important to consider the role that high bacterial load and/or mixed infections (bacterial-bacterial and viral-bacterial) at a young age may play in subsequent risk of OM [10] in a larger cohort of both Aboriginal and non-Aboriginal children [4,22-24].

## Conclusion

This study provides further evidence that early onset of bacterial carriage, and specifically NTHi carriage, increases risk of OM during the first two years of life in Aboriginal children. Furthermore, the study has shown for the first time the importance of early carriage of Mcat as a potential risk factor for OM in non-indigenous Australian children. The results of our study reinforce the need for early interventions to reduce early onset of carriage in order to reduce the burden of OM. Given the association of early NTHi carriage and subsequent OM in Aboriginal children, early administration of pneumococcal-*H*. *influenzae* D protein conjugate vaccine may offer additional benefit. A trial investigating the safety and immunogenicity of such an approach in Aboriginal children, with carriage and OM as secondary outcomes, has recently begun in the NT (http://www.anzctr.org.au/trial_view.aspx?ID=335348). Promotion of hand hygiene among those handling young infants will help reduce URT carriage and hence OM. Improved housing for Aboriginal people to reduce overcrowding is essential as we have previously shown crowding to be the single most important and consistent risk factor for URT carriage [13] which in turn leads to high rates of OM.

## Appendix

### List of investigators and participating institutions

Telethon Institute for Child Health Research (ICHR), Centre for Child Health Research, the University of Western Australia (UWA):

· J Aalberse, A Arumugaswamy, K Carville, D Elsbury, J Evans, J Finucane, C Gordon, P Jacoby, NH de Klerk, D Lehmann, J Mackenzie, A Mason, R Monck, H Moore, F Nichols, K Sivwright, FJ Stanley, A Stokes, W Sun, K Watson, K Wood

Kulunga Research Network, Telethon Institute for Child Health Research:

· J Johnston, D McAullay

The University of Western Australia:

· Harvey Coates, Paediatric Otolaryngologist; J Spencer, C Jeffries-Stokes (also ICHR), P Richmond, paediatricians

Department of Microbiology and Infectious Diseases, PathWest Laboratory Medicine (WA):

· TV Riley (also Microbiology & Immunology, UWA), D Smith (also School of Pathology and Laboratory Medicine, UWA), J Bowman, A Taylor, B Brestovac, G Harnett

Audiologists:

· K Meiklejohn, Country Audiology, Esperance, WA; S Weeks, Professional Hearing Services, Perth, WA

Ngunytju Tjitji Pirni Inc, Kalgoorlie:

· E Edwards, J Carter, A Forrest, G Jones, T Lewis, P McIntosh, J Tamwoy, S Sorian

Bega Garnbirringu Health Service, Kalgoorlie:

· D Dunn, L Dorizzi, S Coleman, R Bonney, P Bonney

University of Canberra:

· A Cripps (also Griffith University, Qld), J Kyd (also Central Queensland University), SM Kyaw-Myint, R Foxwell

Menzies School of Health Research, Darwin, NT:

· AJ Leach, B Harrington, J Beissbarth

Public Health Bacteriology Laboratory, Centre for Public Health Sciences, Brisbane, Qld:

· D Murphy

## Abbreviations

CSOM: Chronic suppurative otitis media; ENT: Ear, Nose and Throat; KOMRP: Kalgoorlie Otitis Media Research Project; Mcat: *Moraxella catarrhalis*; NTHi: Nontypeable *Haemophilus influenzae*; NT: Northern Territory; OM: Otitis media; Pnc: *Streptococcus pneumoniae*; Sa: *Staphylococcus aureus*; URT: Upper respiratory tract; WA: Western Australia.

## Competing interests

D Lehmann, A Cripps and H Coates have been members of the GSK Australia Pneumococcal-*Haemophilus influenzae*-Protein D conjugate vaccine (“PHiD-CV”) Advisory Panel. D Lehmann has received support from Pfizer Australia and GSK Australia to attend conferences. A Cripps and H Coates have been members of the GSK nontypeable *Haemophilus influenzae* Global Advisory Board. A Cripps has received support to attend conferences from GSK Australia and GSK Biologicals. A Leach has received financial support for research and for conference attendance from Pfizer and GSK. A Leach has participated in a GSK-funded Advisory group on CSOM and has undertaken a short term consultancy for GSK.

## Authors’ contributions

WS performed the statistical analysis and drafted the manuscript. PJ provided statistical advice and helped to draft the manuscript. TVR and AJL provided microbiological expertise, contributed to design of study, interpretation of results and preparation of the manuscript. JB carried out primary culture of NPAs. AC participated in the design of the KOMRP, interpretation of the results and preparation of the manuscript. HC and SW carried out clinical examinations, classified the tympanograms and had clinical input into the manuscript. DL conceived and coordinated the KOMRP study and helped to draft the manuscript. All authors have read and approved the final manuscript.

## Authors’ information

Qualifications and current positions:

Wenxing Sun, B Hlth Sci, Research Assistant

Peter Jacoby, MSc, Biostatistician

Thomas V Riley, BAppSc, MAppEpi, PhD, FRCPath, FAAM, FFSc(RCPA),

Professor (The University of Western Australia) and Principal Research Scientist (PathWest Laboratory Medicine (WA))

Jacinta Bowman, BAppSc, Senior Medical Scientist

Amanda Jane Leach, MAgSci, PhD, Leader, Ear Health Research Program

Harvey Coates, MS, FRACS, Clinical Professor and Senior ENT Surgeon

Sharon Weeks, MSc, Audiologist

Allan Cripps, BSc (Hons 1), PhD, FASM, FAIMS, AFCHSM, Pro-Vice Chancellor (Health)

Deborah Lehmann, MBBS, MSc, Principal Research Fellow

## Pre-publication history

The pre-publication history for this paper can be accessed here:

http://www.biomedcentral.com/1471-2334/12/366/prepub
